# DNA metabarcoding reveals ecological patterns and driving mechanisms of archaeal, bacterial, and eukaryotic communities in sediments of the Sansha Yongle Blue Hole

**DOI:** 10.1038/s41598-024-57214-8

**Published:** 2024-03-21

**Authors:** Qingxia Li, Yanli Lei, Tiegang Li

**Affiliations:** 1 Laboratory of Marine Organism Taxonomy and Phylogeny, Qingdao Key Laboratory of Marine Biodiversity and Conservation, Institute of Oceanology, Chinese Academy of Sciences, Qingdao, 266071 China; 2 Key Laboratory of Marine Sedimentology and Environmental Geology, First Institute of Oceanography, Ministry of Natural Resources, Qingdao, 266061 China; 3 Laboratory for Marine Biology and Biotechnology, Qingdao Marine Science and Technology Center, Qingdao, 266237 China; 4 Southern Marine Science and Engineering Guangdong Laboratory (Zhuhai), Zhuhai, 519082 China; 5 Center for Marine Ranching Engineering Science Research of Liaoning, Dalian Ocean University, Dalian, 116023 China; 6 University of Chinese Academy of Sciences, Beijing, 100049 China

**Keywords:** Biodiversity, Community ecology, Molecular ecology, Molecular biology, Environmental sciences

## Abstract

The Sansha Yongle Blue Hole (SYBH) is the world’s deepest marine blue hole with unique physicochemical characteristics. However, our knowledge of the biodiversity and community structure in SYBH sediments remains limited, as past studies have mostly focused on microbial communities in the water column. Here, we collected sediment samples from the aerobic zone (3.1 to 38.6 m) and the deep anaerobic zone (150 m, 300 m) of the SYBH and extracted DNA to characterize the archaeal, bacterial, and eukaryotic communities inhabiting these sediments. Our results showed that the archaeal and bacterial communities were dominated by Thaumarchaeota and Proteobacteria, respectively. The dominant taxa of eukaryotes in different sites varied greatly, mainly including Phaeophyceae, Annelida, Diatomea and Arthropoda. All three examined domains showed clear vertical distributions and significant differences in community composition between the aerobic and anaerobic zones. Sulfide played a prominent role in structuring the three domains, followed by salinity, nitrous oxide, pH, temperature and dissolved oxygen, all of which were positively correlated with the turnover component, the main contributor to beta diversity. Neutral community model revealed that stochastic processes contributed to more than half of the community variations across the three domains. Co-occurrence network showed an equal number of positive and negative interactions in the archaeal network, while positive interactions accounted for ~ 80% in the bacterial and eukaryotic networks. Our findings reveal the ecological features of prokaryotes and eukaryotes in SYBH sediments and shed new light on community dynamics and survival strategies in the special environment of marine blue holes.

## Introduction

Marine blue holes are special geological settings formed in the carbonate banks below the sea level and are dark blue in color^1^. They typically have steep-walled structures and stratified water columns characterized by oxygen deficit and high levels of hydrogen sulfide in deep layers^2,3^. The water exchange between marine blue holes and the open sea is severely restricted, resulting in a relatively stable environment with unique physical–chemical characteristics inside the holes, such as a stratified water column with a strong thermohaline^2^. The special geographical, physical, and chemical features of marine blue holes make them natural laboratories for studying marine biodiversity, biological adaptation, and evolution, as well as the interaction between biotic and abiotic factors^4–6^.

The Sansha Yongle Blue Hole (SYBH; 111°46′06′′ E, 16°31′30′′ N), located within a coral reef of the Yongle Atoll among the Xisha Islands in the South China Sea, is the world’s deepest marine blue hole known so far, with the observed deepest portion at 301.19 m below the local 10-year mean sea level^7^. The three-dimensional (3D) morphology of the SYBH showed that the overall shape of the internal structure resembled a ballet dancer’s shoe with two large transitions at 76–78 m and 158 m, and no water or material exchange with the outside open sea^7^. A comprehensive investigation of the hydrochemical and redox properties of the SYBH revealed the existence of two thermoclines, one at 13–20 m and the other at 70–150 m, which divided the water column into five stratified water layers, and the deep water below 90 m was anaerobic^8^. The existing topography and redox gradients in the water column of the SYBH have increased scientists’ interest in the biodiversity and community structure.

Numerous studies on water column microeukaryotic^9,10^ and prokaryotic plankton^5,11–13^ including bacteria and archaea have expanded our knowledge of the composition, structure, and potential function of the biological communities in the SYBH. The study by Liu et al.^9^ revealed obvious differences in the eukaryotic composition of the water column between the SYBH and the outer reef slope and demonstrated significant effects of turbidity and nitrite concentration on the eukaryotic community structure. Another metabarcoding study suggested that planktonic microeukaryotes in the SYBH were less sensitive to environments but significantly affected by cross-domain biointeraction^10^. Nevertheless, limited information is available concerning the diversity, community structure and spatial distribution of eukaryotic communities in SYBH sediments, and what shapes the eukaryotic assemblages in the sediments remains unclear. For prokaryotes, studies on bacterial community revealed the vertical stratification of community composition in SYBH water column and highlighted the dominance of the phylum Proteobacteria^5,11–13^. Phylogenetic analysis indicated that the SYBH microbial ecosystem was characterized by the presence of unidentified microorganisms in the deep water layer^12^, and a considerable number of new taxa were identified among culturable anaerobic bacteria in SYBH water^13^. However, whether there are also abundant unknown prokaryotic taxa in SYBH sediments remains to be explored.

While previous studies have demonstrated that the geographical isolation and in situ conditions of SYBH contribute to the high microbial and eukaryotic diversity in the water column^5,9,14^, SYBH sediment microorganisms have been poorly studied. This is because, the location of SYBH in the continental slope of the South China Sea, the presence of submerged reefs and the complex geological structures^7,9^ limit access to the bottom of SYBH for sediment collection. The 18S ribosomal DNA study of SYBH sediments indicates high vertical diversity of foraminifera that is influenced by in situ O_2_ conditions and reveals the preference of some foraminiferal taxa for anoxia which may be supported by their ability to denitrify^14^. These findings suggest that SYBH may serve as an excellent natural laboratory to explore additional eukaryotic adaptations associated with anoxia^14^. However, sediment communities have not been well documented in the SYBH, our understanding of the biodiversity and driving factors in SYBH sediments is still limited.

In our study, we collected a total of 12 sediment samples from the aerobic slope (3.1–38.6 m depth) and the anaerobic regime (150 m, and 300 m depth) of SYBH and we performed high-throughput sequencing of 16S and 18S rRNA gene amplicons to examine the prokaryotic (archaea and bacteria) and eukaryotic diversity and distribution in SYBH. We combined our sequencing data results with environmental data to: (1) describe the distribution, diversity, and co-occurrence of archaea, bacteria, and eukaryotes at different oxygen and geochemical regimes of SYBH; (2) compare the differences in community characteristics between the aerobic and anaerobic zones of SYBH; (3) analyze the main environmental factors that influence the composition of the prokaryotic and eukaryotic communities in SYBH sediments.

## Results

### Environmental metadata for SYBH sampling sites

The hydrochemical factors in the water column of SYBH at each sampling site are shown in Table S1. Temperature, pH and the concentrations of dissolved oxygen (DO) and nitrous oxide (N_2_O) generally decreased with depth. Temperature and pH decreased from 30 °C and 8.09 at the surface to 15 °C and 7.49 at the bottom, respectively. DO concentration in the upper water column was ~ 233 μmol L^−1^ and decreased sharply, showing two concentration minima before becoming anoxic below 100 m (Table S1). Contrastingly, salinity, ammonia, dissolved inorganic carbon (DIC), methane, phosphate, silicate, and sulfide increased with depth (from surface to 150 m), maintaining nearly constant levels below 150 m. The concentration of nitrate reached maximum levels at 70 m (5.44 μmol L^−1^) and then sharply decreased to undetectable levels at a depth of 100 m. The concentrations of dissolved organic carbon (DOC), nitrite, particulate organic carbon (POC), and total suspended solid (TSS) fluctuated multiple times from the surface to the bottom, with a relatively stable trend below 200 m. The concentrations of DOC and POC reached a peak at the depth of 150 m.

### Taxonomic diversity and community composition

In the final dataset, we retained 2752 archaeal OTUs representing 223,545 reads, 6266 bacterial OTUs representing 384,644 reads, and 3608 eukaryotic OTUs representing 809,257 reads for downstream analysis (Table S2). The number of bacterial OTUs varied greatly among the 12 SYBH sites (SYBH1-SYBH10 aerobic; SYBH11-SYBH12 anaerobic), ranging from 1903 (SYBH9) to 3734 (SYBH11), with an average of 2864 OTUs per site. The difference in the number of eukaryotic OTUs among the 12 SYBH sites was small, ranging from 1056 (SYBH2) to 1615 (SYBH8), with an average of 1412 OTUs per site. The number of archaeal OTUs ranged from 535 (SYBH8) to 1606 (SYBH5), with an average of 965 OTUs per site. The number of bacterial OTUs and eukaryotic reads in the two anoxic sites (SYBH11 and SYBH12) was higher than that in the 10 aerobic sites (Fig. S1).

**Figure 1:**
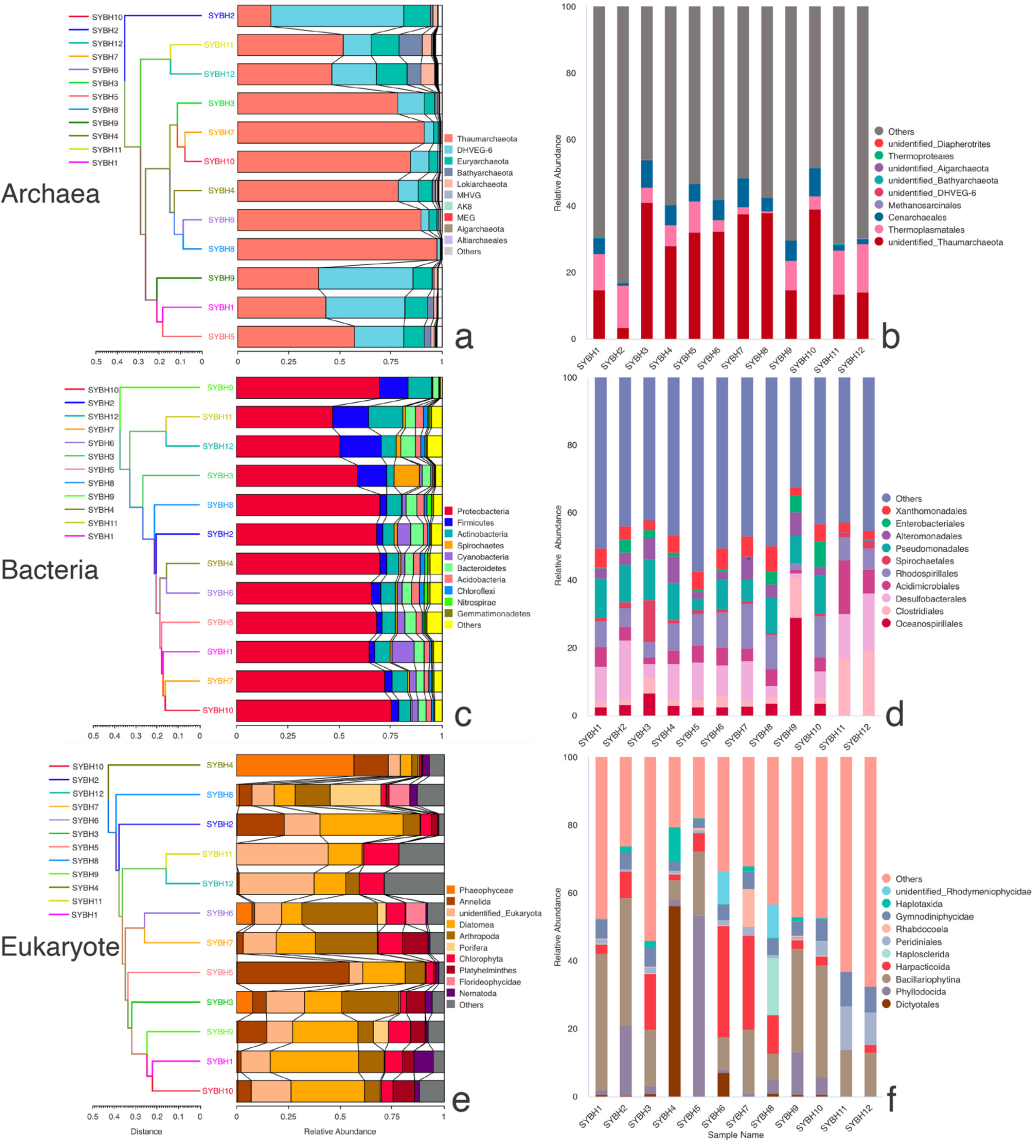
Community composition of archaea, bacteria, and eukaryotes at the phylum level (**a**, **c**, **e**) and order level (**b**, **d**, **f**). The UPGMA analysis was performed based on the Bray–Curtis dissimilarity index at the phylum level.

Our 16S and 18S rRNA gene analysis indicated 16 archaeal, 52 bacterial and 52 eukaryotic phyla. The top three archaeal taxa at the phylum level were Thaumarchaeota, DHVEG-6 and Euryarchaeota (Fig. 1). Thaumarchaeota was the most abundant archaeal phylum accounting for 73.49% of the total archaeal reads and occupied the highest proportion of archaeal reads at 10 SYBH sites. DHVEG-6 had the highest proportion of archaeal reads at sites SYBH2 and SYBH9. Euryarchaeota, Bathyarchaeota and Lokiarchaeota presented higher relative abundance in the two anaerobic sites SYBH11 and SYBH12. The top three bacterial phyla were Proteobacteria, Firmicutes and Actinobacteria. Proteobacteria was the dominant phylum across all SYBH sites, accounting for 63.36% of the total bacterial reads. Firmicutes occupied a high proportion in the two anaerobic SYBH sites. The relative abundance of Actinobacteria in SYBH11 was the highest among the 12 sites. Cyanobacteria occupied higher relative abundance in the two shallow sites SYBH1 and SYBH2. The eukaryotic community varied greatly in composition across the 12 SYBH sites, showing different abundances at the examined sites. Annelida, Diatomea, and Arthropoda were relatively abundant phyla at all sites. The relative abundance of phylum Phaeophyceae at the aerobic site SYBH4 exceeded 50% but was low in other sites. In the anaerobic sites SYBH11 and SYBH12, the eukaryotic community was dominated by the group of unidentified Eukaryota, and only Diatomea, Chlorophyta and Arthropoda had relative abundances greater than 1%. Additionally, the eukaryotic dataset contained 1005 fungal reads and 50 fungal OTUs representing three phyla (Chytridiomycota, Basidiomycota and Ascomycota). The relative abundance of Fungi was less than 0.3% in all SYBH sites (Table S2).

Detailed analysis at the site level revealed clear vertical variations in the alpha diversity of archaea, bacteria and eukaryotes, with different distributions across the 12 sites examined (Fig. S2). Notably, the alpha diversity of bacterial community in the anaerobic zone (SYBH11 and SYBH12) was higher than that in the aerobic zone. The unweighted pair group method with arithmetic mean (UPGMA) cluster analysis of archaeal, bacterial, and eukaryotic communities at the phylum level revealed that the two anaerobic sites, SYBH11 and SYBH12, were clustered on the same branch. The order-level analysis further confirmed that the community composition of SYBH11 and SYBH12 was similar. As the redox regime in the water column changed from aerobic to anaerobic/sulfidic, the relative abundance of some taxa such as Bathyarchaeota, Lokiarchaeota, Firmicutes, Thermoplasmatales, Acidimicrobiales, Clostridiales and Desulfobacterales became higher (Fig. 1). 2570 out of the 2752 archaeal OTUs were not assigned to any archaeal taxon at the order level, indicating that the vast majority of archaeal OTUs (over 93%) remain unidentified at the order level and below. The remaining 182 archaeal OTUs were attributed to 10 archaeal groups at the order level, of which 147 OTUs belong to Thermoplasmatales, five OTUs belong to Methanosarcinales, five OTUs belong to Cenarchaeales and only one OTU belong to Thermoproteales (Table S2). The group of unidentified Thaumarchaeota was represented by five OTUs but accounted for the highest proportion of archaeal reads at the order level (Fig. 1). In the bacterial and eukaryotic datasets, the number of OTUs identified at the order level were 4312 and 2553, accounting for 69% and 71% respectively.

### Correlations between community structure and environmental variables

To examine correlations between the dominant phyla and the environmental variables analyzed in this study, we performed nonparametric Spearman’s correlation analysis (Table 1). The relative abundance of Euryarchaeota was negatively correlated with nitrate (r = − 0.768, *p* < 0.01), nitrite (r = − 0.839, *p* < 0.01), and N_2_O (r = − 0.837, *p* < 0.01), but positively correlated with sulfide (r = 0.640, *p* < 0.05), ammonia (r = 0.723, *p* < 0.01) and methane (r = 0.792, *p* < 0.01). The relative abundance of Proteobacteria exhibited positive correlations with nitrate (r = 0.824, *p* < 0.01), nitrite (r = 0.797, *p* < 0.01) and N_2_O (r = 0.802, *p* < 0.01), but was negatively correlated with sulfide (r = − 0.650, *p* < 0.05), ammonia (r = − 0.754, *p* < 0.01) and methane (r = − 0.844, *p* < 0.01). The relative abundance of Firmicutes was correlated with 11 environmental variables, of which temperature, DO, DOC, POC and pH had negative correlations with Firmicutes, whilst salinity, sulfide, phosphate, silicate, DIC and depth had positive correlations with Firmicutes. The correlations between Firmicutes and temperature and POC were significant (*p* < 0.01), while the remaining nine correlations were not significant (*p* < 0.05). The relative abundance of Arthropoda and Annelida exhibited a negative correlation with sulfide (*p* < 0.05). Arthropoda showed a positive correlation with POC (*p* < 0.05). No significant correlation was detected between the relative abundance of Thaumarchaeota, DHVEG-6, Actinobacteria and Diatomea and the examined environmental variables (*p* > 0.05).

**Table 1:** Results of Spearman’s correlation analysis of dominant taxa at the phylum level in the archaeal, bacterial, and eukaryotic communities and the environmental variables examined, such as temperature (T), DO, ammonia, phosphate, N_2_O, DOC, POC, TSS, and DIC.

	Thaumarchaeota	DHVEG-6	Euryarchaeota	Proteobacteria	Actinobacteria	Firmicutes	Diatomea	Arthropoda	Annelida
T	− 0.182	0.231	− 0.056	0.021	− 0.413	− 0.713^**⁎⁎**^	0.287	0.406	0.490
Salinity	0.091	− 0.147	0.126	0.021	0.510	0.608^**⁎**^	− 0.182	− 0.434	− 0.490
DO	− 0.214	0.277	− 0.018	0.032	− 0.445	− 0.669^**⁎**^	0.389	0.371	0.473
Sulfide	− 0.253	0.124	0.640^**⁎**^	− 0.650^**⁎**^	0.414	0.640^**⁎**^	− 0.253	− 0.591^**⁎**^	− 0.650^**⁎**^
Nitrate	0.521	− 0.408	− 0.768^**⁎⁎**^	0.824^**⁎⁎**^	− 0.113	− 0.085	− 0.007	0.289	0.380
Nitrite	0.566	− 0.462	− 0.839^**⁎⁎**^	0.797^**⁎⁎**^	0.014	− 0.182	− 0.070	0.420	0.343
Ammonia	− 0.404	0.302	0.723^**⁎⁎**^	− 0.754^**⁎⁎**^	− 0.189	0.309	− 0.189	− 0.442	− 0.211
Phosphate	0.141	− 0.197	0.085	0.000	0.465	0.648^**⁎**^	− 0.246	− 0.451	− 0.472
Silicate	0.179	− 0.242	0.049	0.004	0.403	0.701^**⁎**^	− 0.312	− 0.452	− 0.455
N_2_O	0.564	− 0.459	− 0.837^**⁎⁎**^	0.802^**⁎⁎**^	− 0.004	− 0.179	− 0.074	0.427	0.347
Methane	− 0.515	0.410	0.792^**⁎⁎**^	− 0.844^**⁎⁎**^	0.102	0.140	− 0.025	− 0.305	− 0.385
DOC	− 0.343	0.406	0.105	0.000	− 0.476	− 0.608^**⁎**^	0.497	0.168	0.517
POC	0.021	0.035	− 0.268	0.197	0.070	− 0.732^**⁎⁎**^	0.296	0.599^**⁎**^	0.183
TSS	− 0.403	0.414	0.342	− 0.346	− 0.150	− 0.349	0.460	− 0.018	0.089
DIC	0.168	− 0.217	0.063	0.000	0.371	0.699^**⁎**^	− 0.294	− 0.420	− 0.441
pH	− 0.210	0.273	− 0.021	0.027	− 0.427	− 0.671^**⁎**^	0.392	0.364	0.469
Depth	0.168	− 0.217	0.063	0.000	0.371	0.699^**⁎**^	− 0.294	− 0.420	− 0.441

*indicates insignificant correlation (*p* < 0.05); **indicates significant correlation (*p* < 0.01).

**Figure 2:**
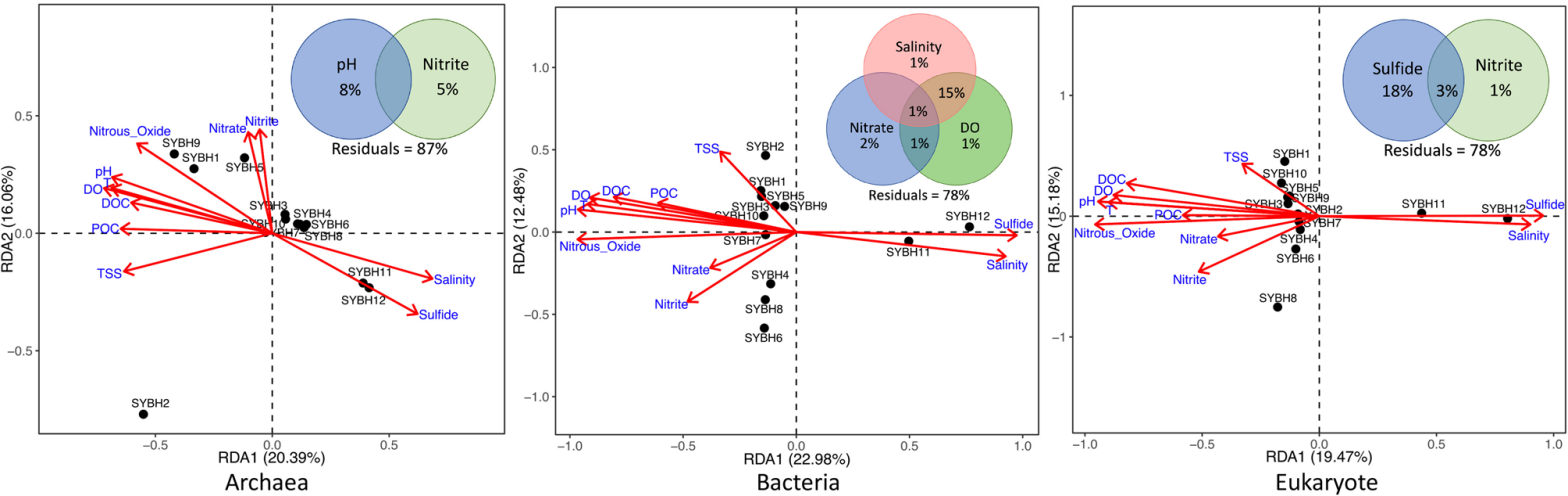
RDA for the archaeal, bacterial and eukaryotic communities to explore the relationships between community structure and environmental variables. Venn diagrams illustrate the relative contribution of the selected variables in shaping archaeal, bacterial, and eukaryotic communities as it was evaluated by VPA.

Redundancy analysis (RDA) results showed that sulfide, salinity and N_2_O had great impacts on the archaeal, bacterial and eukaryotic communities, among which sulfide was the most important factor affecting the three domains in SYBH sediments. The strongest influence of sulfide was at the two anaerobic sites, SYBH11 and SYBH12. Nitrate, nitrite, TSS and POC had relatively weak effects on the distribution of archaea, bacteria and eukaryotes (Fig. 2), but the effects were different for the three domains. As an example, BIOENV analysis revealed that nitrite and pH could best explain the archaeal community structure. In particular, the variation partitioning analysis (VPA) results indicated that nitrite and pH explained 13% of archaeal community variation. Environmental variables that correlated with the bacterial community assemblages included salinity, nitrate, and DO, explaining 22% of the bacterial community variation (Fig. 2). For eukaryotes, sulfide influenced the community structure and together with nitrite, explained 22% of community variation (Fig. 2).

Partitioning beta-diversity using multiple-site dissimilarity measures showed that the turnover component (βsim) contributed at least eight times more to the Sørensen dissimilarity (βsor) of the archaeal, bacterial, and eukaryotic communities than the nestedness component (βsne). This indicates that the variation in species composition among sites was affected by species replacement between sites rather than species loss from site to site (Table 2).

**Table 2:** Results of partitioning multiple-site beta diversity (βsor) into turnover component (βsim) and nestedness component (βsne) by using Baselga’s method.

	βsor	βsim	βsne
Archaea	0.8391	0.7504	0.0887
Bacteria	0.7411	0.6883	0.0528
Eukaryote	0.7630	0.7269	0.0361

**Figure 3:**
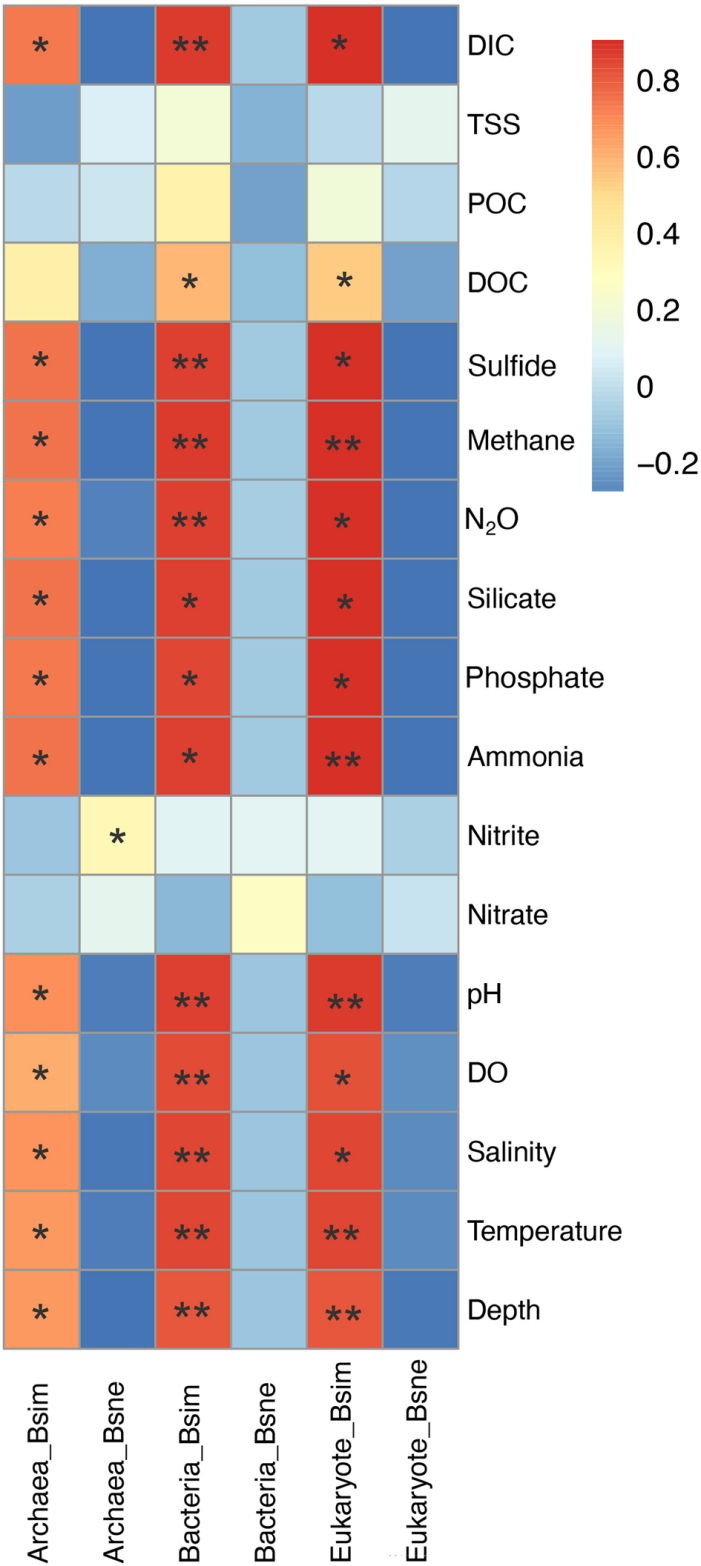
The relationships between two β-diversity components (βsim and βsne) and the environmental variables which were determine by Mantel test based on Pearson’s correlations. * indicates insignificant correlation (*p* < 0.05); ** indicates significant correlation (*p* < 0.01).

Mantel test between the pairwise dissimilarity metrics and the Euclidean distance of environmental factors further revealed that the turnover component (βsim) of Sørensen dissimilarity of the archaeal, bacterial, and eukaryotic communities was influenced by a wide range of environmental variables, including water depth, temperature, salinity, DO, pH, ammonia, phosphate, silicate, N_2_O, methane, sulfide and DIC (Fig. 3). There were nine significant correlations in the bacterial community, and five significant correlations in the eukaryotic community. Water depth, temperature, pH and methane had significant effects on the βsim of both bacterial and eukaryotic communities (*p* < 0.01). Furthermore, the βsim of the bacterial and eukaryotic communities was correlated with DOC (*p* < 0.05). Consistent with the RDA results, the βsim of the three domains was not correlated with nitrate, nitrite, POC and TSS. The nestedness component (βsne) of the three domains was not significantly correlated with environmental variables.

### The community assembly and co-occurrence network analysis

**Figure 4:**
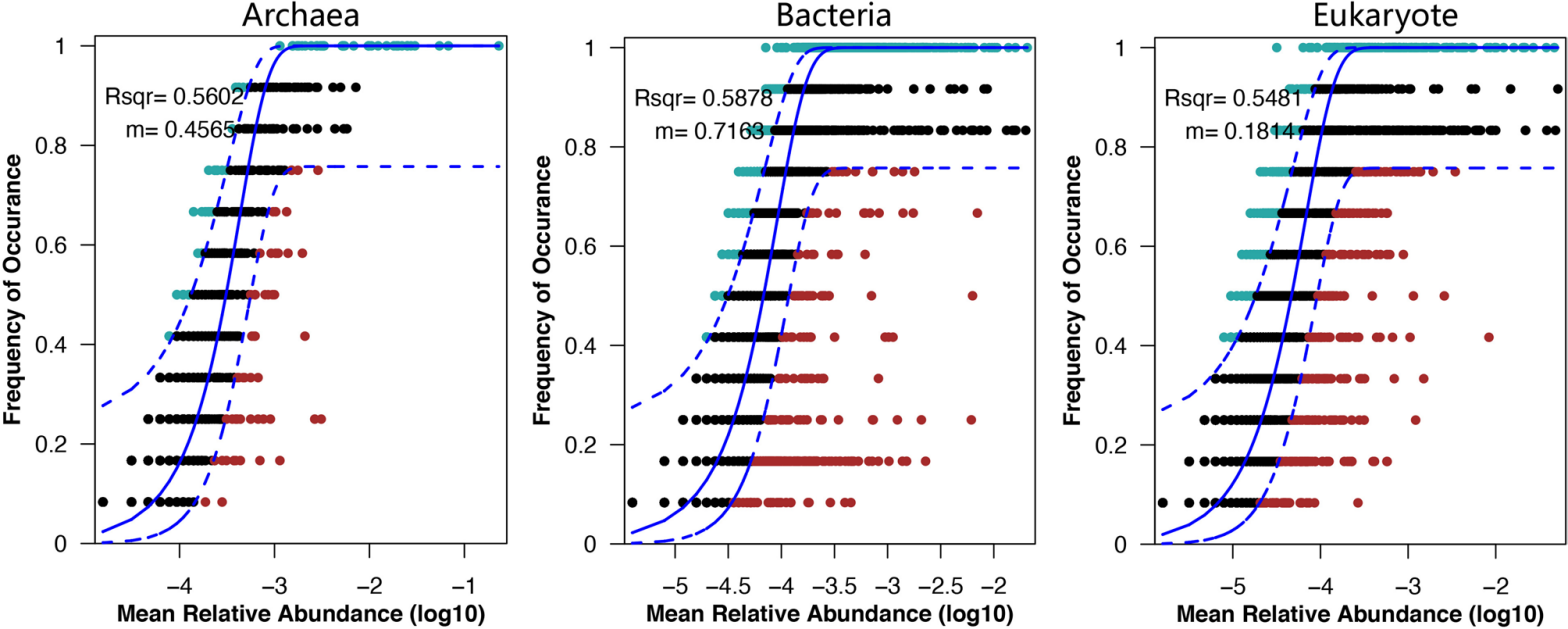
Fitting of a neutral community model (NCM) to the community assemblage at the OTU level. The frequencies of occurrence of archaea, bacteria and eukaryotes were predicted. The parameters *R*^2^ and *m* represent the fit to the neutral model and the immigration rate, respectively.

The Sloan neutral community model showed that the *R*^2^ values in the archaeal, bacterial, and eukaryotic communities were 56.02%, 58.78% and 54.81%, respectively (Fig. 4). These results indicated that stochastic processes contributed to more than half of the community variations across the three domains. The *m* value in the bacterial community was the largest (*m* = 0.7163), followed by that of the archaeal community (*m* = 0.4565), and the *m* value in the eukaryotic community was the smallest (*m* = 0.1814), indicating that the species dispersal of bacteria was relatively high, while the species dispersal in the eukaryotic community was limited.

**Figure 5:**
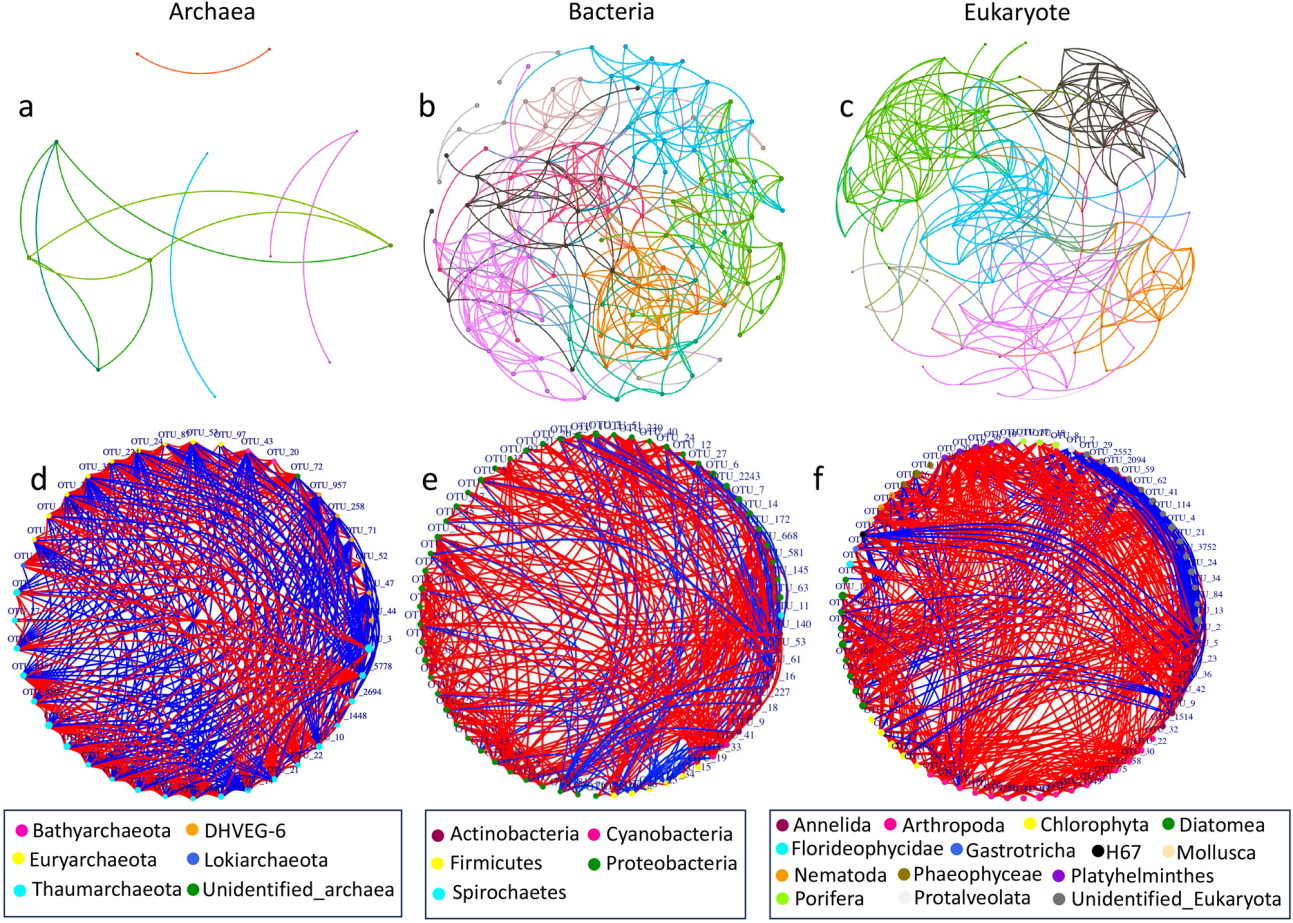
Co-occurrence networks at the genus level (**a**-**c**) and at the OTU level (**d**-**f**). The networks at the genus level were colored according to the modularity class. In the networks based on OTUs, red lines represent positive interactions among OTUs, and the blue lines represent negative interactions among OTUs.

The bacterial and eukaryotic genera with a relative abundance of more than 0.5% and all archaeal genera were selected to generate the co-occurrence network in the SYBH (Fig. 5). The bacterial network was more complex than the archaeal network and eukaryotic network at the genus level (Fig. 5a-c). The numbers of vertices in the archaeal, bacterial, and eukaryotic networks at the genus level were 12, 121, and 87, respectively, and their edge numbers were 13, 385, and 291, respectively. The co-occurrence networks constructed based on OTU abundances reflected that the number of positive interactions was close to that of negative interactions (195 vs. 177) in the archaeal network. In the bacterial and eukaryotic networks, most of the interactions were positive, accounting for 82% and 80%, respectively (Fig. 5d-f). In the archaeal network, most of the keystone OTUs were derived from Thaumarchaeota and Euryarchaeota. Thaumarchaeota had negative interactions with Euryarchaeota and DHVEG-6, and Euryarchaeota showed positive interactions with Lokiarchaeota and Bathyarchaeota. Because only 7% of archaeal OTUs were assigned below the order level, more interactions may occur between archaeal taxa in SYBH, which warrants further investigation. In the bacterial network, most of the keystone OTUs belonged to the phylum Proteobacteria, which had strong positive interactions with Cyanobacteria. Strong negative interactions were observed between Firmicutes and Cyanobacteria. In the eukaryotic network, most of the highly connected OTUs were derived from the Unidentified Eukaryota, indicating putative importance of the unidentified fraction of eukaryotes in SYBH sediments.

## Discussion

As the deepest marine blue hole known in the world, Sansha Yongle Blue Hole has high scientific research values and has attracted widespread attention. Our study presented the first DNA metabarcoding dataset covering the three domains of cellular life (archaea, bacteria, and eukaryotes) from the SYBH sediment samples. Further it provides a comprehensive analysis of the molecular diversity, community structure and assembly mechanism, and indicates the role of environmental factors in shaping the prokaryotic and eukaryotic communities in the SYBH.

Marine blue holes are generally characterized by steep physicochemical gradients within geographically restricted areas^1,13^, creating conditions for new, unidentified microbial lineages to exist^6^. High-quality metagenome-assembled genomes (MAGs) retrieved recently from the Amberjack Hole (Gulf of Mexico blue hole) showed that microbial communities were dominated by understudied microbial taxa, indicating high levels of novel microbial lineages in the Amberjack Hole^6^. Similarly, some studies indicated that the microbial ecosystems in SYBH water column were also characterized by unidentified taxa^11,12^. A recent study on microbial community in SYBH water samples recovered high-quality MAGs belonging to various uncultivated lineages, reflecting the high novelty of the SYBH microbiome^15^. In this study, 29% of eukaryotic OTUs, 31% of bacterial OTUs, and more than 93% of archaeal OTUs remained unidentified at the order level, indicating the existence of a large number of undocumented prokaryotic and eukaryotic taxa in SYBH sediments. The high percentages of undocumented taxa in our study agree with the high number of unidentified lineages in the water column of SYBH^11,12,15^, which indicates that microorganisms inhabiting SYBH (both sediment and water column) are underrepresented in public databases. This hinders adequate taxonomic identifications of the in situ prokaryotic and eukaryotic communities in SYBH. The presence of high levels of novel lineages in the SYBH is not surprising, as this phenomenon has also been observed in other marine habitats. Recent studies have revealed novel lineages of Southern Ocean deep-sea foraminifera^16^ and high genetic novelty of foraminifera on Western Pacific seamounts^17,18^. The unique environmental characteristics of habitats such as marine blue holes and seamounts provide conditions for the emergence of new species.

This study also revealed the community composition of the three domains at the phylum and order levels. To summarize, the bacterial community in SYBH sediments was dominated by Proteobacteria (Fig. 1), which is consistent with the results of bacterial communities in SYBH water^5,11–13^. The relative abundance of Proteobacteria in deep anaerobic sites was lower than that in upper aerobic sites (Fig. 1). This may be mainly related to nutrient availability, as the relative abundance of Proteobacteria was significantly positively correlated with nitrate and nitrite, which were undetectable below 100 m. As for the archaeal community, the study by He et al.^5^ revealed the dominance of Euryarchaeota in SYBH water, but a recent study by Chen et al.^19^ showed that Nanoarchaeota and Agenigamarchaeota dominated the archaeal community in SYBH water. This divergence in results may be due to differences in the primers used, but the exact cause deserves further exploration. Different from the above research results^5,19^, a study on the archaeal communities in the deep-sea sediments of the South China Sea showed that archaeal communities in sediments were dominated by Thaumarchaeota (55%)^20^. Our results on the fraction of archaeal community that were able to assign taxonomy confirmed the dominance of Thaumarchaeota, which dominated the archaeal community at 10 SYBH sites with the relative abundance ranging from 43 to 98% (Fig. 1). DHVEG-6 was the second most abundant taxon in the archaeal community at the phylum level, with the relative abundance ranging from 2 to 65% across all SYBH sites. The dominant taxa of eukaryotic community varied greatly among the 12 SYBH sites. In aerobic sites, the dominant eukaryotic taxa included Annelida, Arthropoda and Diatomea, while the eukaryotic communities in anaerobic sites were dominated by unidentified Eukaryota. Notably, in the study of SYBH water column by Chen et al.^19^, a total of 7888 amplicon sequence variants (ASVs) were identified from the fungi dataset. However, only a small fraction of fungi (50 fungal OTUs representing 1005 reads) were detected in our eukaryotic dataset (Table S2). The fact that our dataset did not recruit many fungal OTUs may be caused by primer specificity, as we used general 18S rRNA primers rather than ITS primers.

Vertical variation is a typical distribution pattern for biological communities in the water column of the SYBH which has been observed in the eukaryotic^14,19^ and prokaryotic communities^5,13,21^. The water column in SYBH is generally divided into an aerobic surface layer (at depths of 0–70 m), an intermediate layer (at depths of 70–100 m) and an anoxic bottom layer (below 100 m) along the depth gradient^8^. These different water layers have different microbial compositions^5^ and redox processes^8^. Although we did not obtain sediment samples within chemocline (at depths of 70–100 m), our results still clearly reveal vertical changes in prokaryotic and eukaryotic communities in SYBH sediments with water depth. In the aerobic layer close to surface and withing the euphotic zone (3.1–38.6 m), Cyanobacteria capable of oxygen-producing photosynthesis had the highest relative abundance in the shallowest site SYBH1 where the DO concentration was the highest. As the redox regime changed from aerobic to anaerobic/sulfidic, the relative abundance of some taxa became higher, such as Bathyarchaeota, Lokiarchaeota, Firmicutes, Thermoplasmatales, Acidimicrobiales, Clostridiales and Desulfobacterales (Fig. 1). These taxa may be associated with crucial biogeochemical processes and play key roles in maintaining the stability of ecosystems in anaerobic zones. Bathyarchaeota are a globally distributed archaeal phylum and important members of global biogeochemical cycles that are shown to highly abundant in anoxic sediments and are considered to utilize a wide range of labile and recalcitrant carbon sources^22^. Firmicutes, a group of syntrophic bacterial taxa, can participate in biogeochemical cycles by degrading organic polymers and lignocellulosic plant material to H_2_, or converting small molecular compounds to H_2_ and acetate^12^. Methanogenic archaea (phylum Euryarchaeota, order Methanosarcinales) were also identified from nine SYBH sites, and their relative abundance was higher in anaerobic zones with high levels of methane concentration (Table S2). Our findings highlight differences in community composition between the aerobic and anaerobic zones of the SYBH and demonstrate that SYBH is a natural laboratory for understanding ecosystem function under oxygen deficiency across redox gradients.

As mentioned, the relative abundance of some prokaryotic and eukaryotic taxa varied greatly between SYBH sites, such as Thaumarchaeota, DHVEG-6, Actinobacteria, Annelida, Arthropoda and Diatomea. Inferring from the results of our analysis, the distinct variations in relative abundance of these taxa between sites may be determined not only by environmental factors but also by strong biotic interactions. A growing number of studies emphasize that biotic interactions play an important role in influencing community diversity^23^, driving key ecological processes^24^, and governing species distributions at macroecological scales^25^. Co-occurrence network analysis is a useful approach for exploring complex interactions within communities^26^. The co-occurrence networks in this study indicated that the above-mentioned taxa had complex interaction networks (Fig. 5). For example, Thaumarchaeota had negative interactions with Euryarchaeota and DHVEG-6, Annelida exhibited negative interactions with Chlorophyta, and Diatomea showed positive interactions with Chlorophyta. Members of Thaumarchaeota, Arthropoda and Annelida also showed a high proportion of intra-phylum interactions. Chen et al.^10^ suggested that abiotic factors played a minor role in shaping microeukaryotic plankton community in SYBH, and interspecies cooperation might be one of the ecological strategies. Here, co-occurrence networks suggest that biotic interactions (e.g., mutualism, commensalism, synergism) may play an important role in the distribution of these dominant taxa of prokaryotic and eukaryotic communities in SYBH sediments. Furthermore, co-occurrence network analysis can provide new insights into keystone species in communities^26^. In this study, the keystone OTUs in the archaeal, bacterial, and eukaryotic networks were mainly derived from Thaumarchaeota, Proteobacteria and unidentified Eukaryota, respectively, indicating that they potentially exerted a considerable impact on the community. At the OTU level, most correlations in bacterial and eukaryotic networks were positive (Fig. 5), consistent with previous research^5^, reflecting the widespread beneficial interspecific interactions such as mutualism, commensalism, and facilitation^27^ in the bacterial and eukaryotic communities. In the archaeal network, the number of positive correlations was close to the number of negative correlations (Fig. 5), which indicated that negative interspecific interactions (e.g., competition) and the aforementioned positive interspecific interactions played roles in shaping community characteristics.

The mechanisms that shape microbial community structure have always been a key issue in understanding community dynamics in the SYBH. The results of He et al.^5^ suggested that temperature and nitrate concentration had significant contribution to the heterogeneous distribution of major bacterial clades and salinity explained most variations of the archaeal communities in SYBH water column. Similarly, Zhang et al.^12^ illustrated the significant correlations between the bacteria and environmental elements of DO, temperature, salinity, pH, sulfur and nutrient. However, the above research results are all focused on the microbial community in SYBH water. The RDA results in our study illustrated that sulfide, salinity, N_2_O, pH, DO and temperature had great impacts on the archaeal, bacterial and eukaryotic communities, among which sulfide was the most prominent factor affecting the community structure in SYBH sediments (Fig. 2). Finally, our beta diversity metrics may reflect spatial turnover and nestedness of assemblages, caused by species replacement and species loss, which are common traits that influence biodiversity^28^. By applying Baselga's approach^29^, our results show that the turnover component (βsim) is the main contributor to beta diversity (βsor) of the archaeal, bacterial and eukaryotic communities in SYBH sediments (Table 2). This indicates that the overall patterns of multiple-sites dissimilarity of the three domains in SYBH sediments are driven by species replacement rather than species loss, which may be the consequence of either environmental sorting or spatial and historical constraints^30^. The Mantel test further revealed that the turnover component (βsim) of the three domains was influenced by a wide range of environmental variables, including sulfide, salinity, N_2_O, pH, DO and temperature (Fig. 3). Compared with bacterial and eukaryotic communities, the βsim of archaeal community seems to be less affected by environmental variables. Quantifying the relative importance of deterministic and stochastic processes that shape microbial community assembly is considered a central challenge in ecology^31,32^, and also for SYBH. The NCM proposed by Sloan et al.^33^ is particularly useful in quantifying the importance of neutral processes and has been employed in bacterial community^34–36^, eukaryotic community^37^ and microeukaryotic community^38,39^. Although stochastic processes seem to influence planktonic microeukaryotes and deterministic processes dominate in prokaryotic plankton in the SYBH^10^, our data from SYBH sediments indicate that stochastic processes appear to shape the sediment communities. Our results show that stochastic processes contributed to ~ 55% of the variations in archaeal, bacterial, and eukaryotic communities in SYBH sediments (Fig. 4), indicating that stochastic processes might play more important roles than deterministic processes in the sedimental communities. Overall, the SYBH supports an interesting ecosystem that warrants further investigation in the future to decipher the metabolic capabilities of the identified taxa. This will facilitate our understanding of the biogeochemistry that exists in marine blue holes and how it is shaped by the in situ microbial communities.

## Materials and methods

### Sample collection

Sediment samples were collected from 12 sites at different water depths in the SYBH on board R/V CHANGHE OCEAN on May 17–28, 2017. Among the 12 sites, 10 sites were located in the oxygenated mixed layer with water depth ranging from 3.1 to 38.6 m, and the sediment samples from these 10 sites were recovered by scuba diving. The other two sediment samples were collected from the anaerobic layer at 150 and 300 m respectively with a ROV. All sediment samples were stored in liquid nitrogen on board until further processing. At each site, the hydrochemical properties were measured in the water column at the depth where sediment samples were collected. The supplementary information of the sediment samples can be found in Table S1 and Figure S2 of Li et al.^14^.

### DNA extraction, PCR amplification and illumina sequencing

DNA was extracted from a 0.25 g sediment using the DNeasy PowerSoil kit (QIAGEN, Germany) following the manufacturer's instructions. One negative control without sediment was incorporated for each extraction session and three DNA replicates were extracted from each sample.

The V3-V4 hypervariable region of the bacterial 16S rRNA gene was amplified using the primers 341F (5’-CCTAYGGGRBGCASCAG-3’) and 806R (5’-GGACTACNNGGGTATCTAAT-3’)^40^. The V4 region of 18S rRNA gene was amplified using the primers 528F (5’-GCGGTAATTCCAGCTCCAA-3’) and 706R (5’-AATCCRAGAATTTCACCTCT-3’)^41^ for eukaryote. The V4 region of the archaeal 16S rRNA gene was amplified using the primers Arch519F (5’- CAGCCGCCGCGGTAA-3’) and Arch915R (5’- GTGCTCCCCCGCCAATTCCT-3’)^42^. Forward and reverse primers were tagged with six nucleotide-long sequences appended at their 5’-end to multiplex the PCR products in a unique sequencing library. All PCR reactions were performed in a total volume of 30 μL containing 15 μL of Phusion High-Fidelity PCR Master Mix (New England Biolabs, Beverly, MA, USA), 0.2 μM of forward and reverse primers, and about 10 ng template DNA. Thermal cycling consisted of a pre-denaturation at 98 °C for 1 min, followed by 30 cycles of denaturation at 98 °C for 10 s, annealing at 50 °C for 30 s, and extension at 72 °C for 30 s, followed by a final extension step at 72 °C for 5 min. One PCR blank control without DNA was included during each amplification. Each PCR product was mixed with same volume of 1 × loading buffer, and electrophoresis was performed on 2% agarose gel for detection. The triplicated PCR products of one sample were mixed in equidensity ratios and the mixed PCR products were purified with Qiagen Gel Extraction Kit (Qiagen, Germany).

Sequencing libraries were generated using TruSeq DNA PCR-Free Sample Preparation Kit (Illumina, USA) following manufacturer's recommendations. The library quality was assessed by the Qubit 2.0 Fluorometer (Thermo Scientific) and Agilent Bioanalyzer 2100 system. The library was sequenced on an Illumina HiSeq2500 platform and 250 bp paired-end reads were generated at the Novogene Bioinformatics Technology Co., Ltd (Beijing). The sequencing reads were submitted to the Sequence Read Archive under accession number PRJNA998750.

### Sequence processing

Raw paired-end reads were de-multiplexed to samples based on their unique barcode and truncated by cutting off the barcode and primer sequences. They were merged using FLASH^43^, and the spliced sequences were called raw reads. Quality filtering on the raw reads were performed under specific filtering conditions according to the QIIME^44^ quality controlled process and low-quality sequences were filtered out. Chimera sequences were removed from reserved sequences using UCHIME algorithm. High-quality sequences were clustered into operational taxonomic units (OTUs) at a 97% similarity level using UPARSE pipeline^45^. The taxonomic assignment of bacterial OTUs and archaeal OTUs was performed using the Mothur method and the SILVA database (version 132)^46^, and the eukaryotic OTUs were taxonomically classified using the Ribosomal Database Project (RDP) classifier and SILVA database (version 132)^46^. After taxonomic assignment, we excluded the OTUs that could not be assigned to the target community.

### Diversity and statistical analysis

Before calculating alpha and beta diversity, we normalized the data of each sample using a standard of reads number corresponding to the sample with the least reads using the R package vegan. The standard of reads number for archaea, bacteria and eukaryotes was 5340, 21,029 and 52,561, respectively. Alpha diversity estimated by Chao1 and Shannon index of the archaeal, bacterial, and eukaryotic communities were calculated on the normalized data with R packages vegan and picante. Community composition of archaea, bacteria, and eukaryotes at the phylum and order levels were visualized in Fig. 1 with the R package ggplot2^47^. The UPGMA analysis was performed based on the Bray–Curtis dissimilarity index at the phylum level (Fig. 1).

Preliminary detrended correspondence analysis (DCA) was used to evaluate whether RDA or CCA was more suitable for exploring the relationships between community and environmental variables^48^. Preliminary DCA results revealed that the longest gradient length of the archaeal, bacterial, and eukaryotic communities was 2.69, 3.37, and 3.95 (> 3 standard deviations), respectively. Therefore, RDA was chosen for archaea, bacteria and eukaryotes to assess the relationships between community structure and environmental variables^49,50^. To improve normality and homoscedasticity, all environmental factors were log (x + 1) transformed before analysis. BIOENV and VPA analysis in R package vegan were used to identify the major environmental variables and evaluate their relative contribution in driving distribution patterns across the three domains (Fig. 2).

### Partitioning of beta diversity

The beta-diversity as Sørensen index (βsor) of the archaeal, bacterial, and eukaryotic communities were partitioned into two components, turnover (βsim) and nestedness (βsne), by applying Baselga’s approach (Table 2). The multiple-site measures of compositional dissimilarity across all sites and pairwise between-site dissimilarity matrices were computed from the presence-absence matrix using the function beta.multi and beta.pair of the R package “betapart”, respectively^29^. The relationships between the environmental variables and the βsim and βsne were determine by Mantel test based on Pearson’s correlations (Fig. 3).

### Community assembly process

The potential importance of stochastic processes to community assembly was evaluated using the Sloan’s NCM, which predicted the relationship between the occurrence frequency of OTUs and their relative abundance in the metacommunity^33^. The model used here is an adaptation of the neutral theory^51^ adjusted to fit large microbial populations, and the analysis was performed using non-linear least-squares fitting with the R package minpack.lm^52^. The parameters *R*^2^ and *m* represent the fit to the neutral model and the immigration rate, respectively (Fig. 4). Calculation of 95% confidence intervals around all fitting statistics was done by bootstrapping with 1000 bootstrap replicates.

### Co-occurrence network construction

Co-occurrence network analysis was conducted at the genus level and OTU level using R packages psych, igraph and Hmisc and visualized in Fig. 5. At the genus level, the bacterial genus and eukaryotic genus with a relative abundance of more than 0.5% and all archaeal genera were selected to generate the co-occurrence patterns (Fig. 5a–c). A Spearman’s coefficient of greater than 0.7 and a significance level of less than 0.05 indicated a significant correlation. The network diagram was generated by Gephi software^53^. At the OTU level, only the OTUs with a relative abundance of more than 3% were selected to generate the co-occurrence patterns to show the interactions among OTUs (Fig. 5d–f). A Spearman’s coefficient of greater than 0.6 and a significance level of less than 0.05 indicated a significant correlation.

### Supplementary Information

Supplementary Figures.

Supplementary Table S1.

Supplementary Table S2.

## Data Availability

The sequencing data can be downloaded from the NCBI Sequence Read Archive (SRA) under accession number PRJNA998750 (https://www.ncbi.nlm.nih.gov/bioproject?term=PRJNA998750&cmd=DetailsSearch).
