# Supplementary material for: DNA metabarcoding reveals ecological patterns and driving mechanisms of archaeal, bacterial, and eukaryotic communities in sediments of the Sansha Yongle Blue Hole
Source: Sci Rep. 2024 Mar 21;14:6745. doi: 10.1038/s41598-024-57214-8 (PMC10954614; doi:10.1038/s41598-024-57214-8)
Supplement: Supplementary file 1 — Supplementary Figures. [file 41598_2024_57214_MOESM1_ESM.docx]

DNA metabarcoding reveals ecological patterns and driving mechanisms of archaeal, bacterial, and eukaryotic communities in sediments of the Sansha Yongle Blue Hole

**Qingxia Li^1^, Yanli Lei^1,3,4*^, Tiegang Li^2*^**

^1^ Laboratory of Marine Organism Taxonomy and Phylogeny, Qingdao Key Laboratory of Marine Biodiversity and Conservation, Institute of Oceanology, Chinese Academy of Sciences, Qingdao 266071, China

^2^ Key Laboratory of Marine Sedimentology and Environmental Geology, First Institute of Oceanography, Ministry of Natural Resources, Qingdao 266061, China

^3^ Southern Marine Science and Engineering Guangdong Laboratory (Zhuhai), Zhuhai 519082, China

^4^ University of Chinese Academy of Sciences, Beijing 100049, China

*Corresponding authors: Yanli Lei: leiyanli@qdio.ac.cn; Tiegang Li: [tgli@fio.org.cn](mailto:tgli@fio.org.cn)

**Supplementary Figures**

**Fig. S1.** The number of reads and OTUs assigned to archaea, bacteria, and eukaryotes in the 12 SYBH sites.

**Fig. S2.** The Shannon index and Chao1 of the archaeal, bacterial, and eukaryotic communities in the 12 SYBH sites.
